# KIF14 silencing suppresses cell proliferation and enhances chemosensitivity by modulating 53BP1 nucleocytoplasmic shuttling in non-WNT/non-SHH medulloblastoma

**DOI:** 10.1038/s41420-026-03252-2

**Published:** 2026-07-14

**Authors:** Huangyi Fang, Tao Xu, Wei Wen, Shixuan Xiang, Yusong Zhang, Donghao Qin, Anjie Lu, Yutuo Zheng, Lizhong Wang, Regina Chizi Tunje, Zejiang Hong, Siyu Wang, Xiufeng Huang, Hansong Sheng, Jian Lin

**Affiliations:** 1 Department of Neurosurgery, Wenzhou Municipal Key Laboratory of Neurodevelopmental Pathology and Physiology, The Second Affiliated Hospital and Yuying Children’s Hospital of Wenzhou Medical University, Wenzhou, China; 2 Alberta Institute, Wenzhou Medical University, Wenzhou, China; 3 The First People’s Hospital of Jiashan, Jiaxing, China

**Keywords:** Paediatric cancer, CNS cancer

## Abstract

Medulloblastoma (MB) is the most common malignant pediatric brain tumor. The non-WNT/non-SHH subtypes (Groups 3 and 4) exhibit high genetic heterogeneity and limited therapeutic options. Kinesin family member 14 (KIF14) is selectively expressed during nervous system development. However, its role in non-WNT/non-SHH MB remains poorly understood. In this study, analysis of the GEO database revealed that high KIF14 expression correlates with poor prognosis in patients with non-WNT/non-SHH MB. Silencing KIF14 in MB cells impaired cytokinesis, leading to p53-mediated cell cycle arrest and apoptosis. Mechanistically, KIF14 knockdown disrupted the nucleocytoplasmic shuttling of the p53-binding protein 53BP1, resulting in its nuclear accumulation. This shift inhibited homologous recombination (HR)-mediated repair of DNA double-stranded breaks (DSBs), thereby enhancing cisplatin-induced DNA damage. In vitro experiments and intracranial orthotopic xenograft models confirmed that KIF14 silencing potentiates the antitumor efficacy of cisplatin treatment. Overall, these findings highlight the critical role of KIF14 in promoting cell proliferation and DNA repair in non-WNT/non-SHH MB and suggest that targeting KIF14 may represent a novel therapeutic strategy to enhance the efficacy of DNA-damaging agents in this challenging MB subtype.

## Introduction

Medulloblastoma (MB), an embryonic tumor of the cerebellum, is the most common malignant high-grade brain tumor in children [1]. Advances in microarray and genomic sequencing technologies led to the classification of MB into four molecular subgroups in 2012: WNT, SHH, Group 3, and Group 4 [2, 3]. The WNT and SHH subgroups are characterized by constitutive activation of the Wingless and Sonic Hedgehog signaling pathways, respectively. In contrast, Group 3 and Group 4 MBs are the most complex, representing the most genetically heterogeneous and least known subtypes [2, 4]. In the latest WHO classification of central nervous system tumors, these two groups are now collectively referred to as non-WNT/non-SHH MB [5]. Unlike WNT and SHH MBs, over 5%–10% of patients with non-WNT/non-SHH MB harbor no identifiable somatic driver mutation, posing significant challenges for the development of targeted therapies and innovative treatment strategies [6].

One promising strategy for cancer therapy is to target molecular pathways altered by driver mutations. This strategy has led to the development of targeted treatment of SHH MB, including various SHH pathway inhibitors such as vismodegib and erismodegib [7, 8]. However, due to the high genetic heterogeneity of the non-WNT/non-SHH MB, no effective pathway-specific inhibitors have been identified for this subgroup. Although the origin of the different MB subtypes remains unclear, MB cells are known to share molecular characteristics with granule neuron progenitors [9]. Primary microcephaly (MCPH) is a neurodevelopmental disorder marked by a significant reduction in brain volume [10, 11]. Genes associated with MCPH regulate progenitor cell division during neural development and are also essential for malignant proliferation of brain tumor cells [12, 13]. Notably, these genes are rarely expressed in postmitotic cells of the central nervous system [13], making them attractive and potentially selective therapeutic targets for MB.

KIF14 is a microtubule-dependent molecular motor protein. Recessive mutations in KIF14 are linked to MCPH20 syndrome, which is characterized by telencephalic and cerebellar hypoplasia [14]. KIF14 contains an internal motor domain with a strong affinity for microtubules and plays a critical role in cytokinesis by facilitating intracellular bridge cleavage during mitosis [15]. Its expression is low in postnatal adult brain tissues but markedly elevated in placental and fetal brain tissues [16]. Notably, KIF14 has been identified as a potential oncogene located in the chromosome 1q region and is expressed at levels 22–79-fold higher in MB cells compared to fetal brain tissue [16]. Knockdown of KIF14 in MB cells inhibited cell proliferation and induced apoptosis, likely due to delayed mitosis that triggers genotoxic stress [17].

In this study, we found that KIF14 expression was significantly elevated in non-WNT/non-SHH MB compared to normal cerebellar tissue, and high KIF14 expression was associated with poor patient survival. Silencing KIF14 activated the p53 signaling pathway and impaired DNA damage repair by disrupting the nucleocytoplasmic shuttling of 53BP1. These effects led to suppressed tumor cell proliferation and increased sensitivity to the DNA-damaging agent cisplatin.

## Results

### Elevated KIF14 expression predicts poor prognosis and facilitates diagnosis in non-WNT/non-SHH MB

**Fig. 1:**
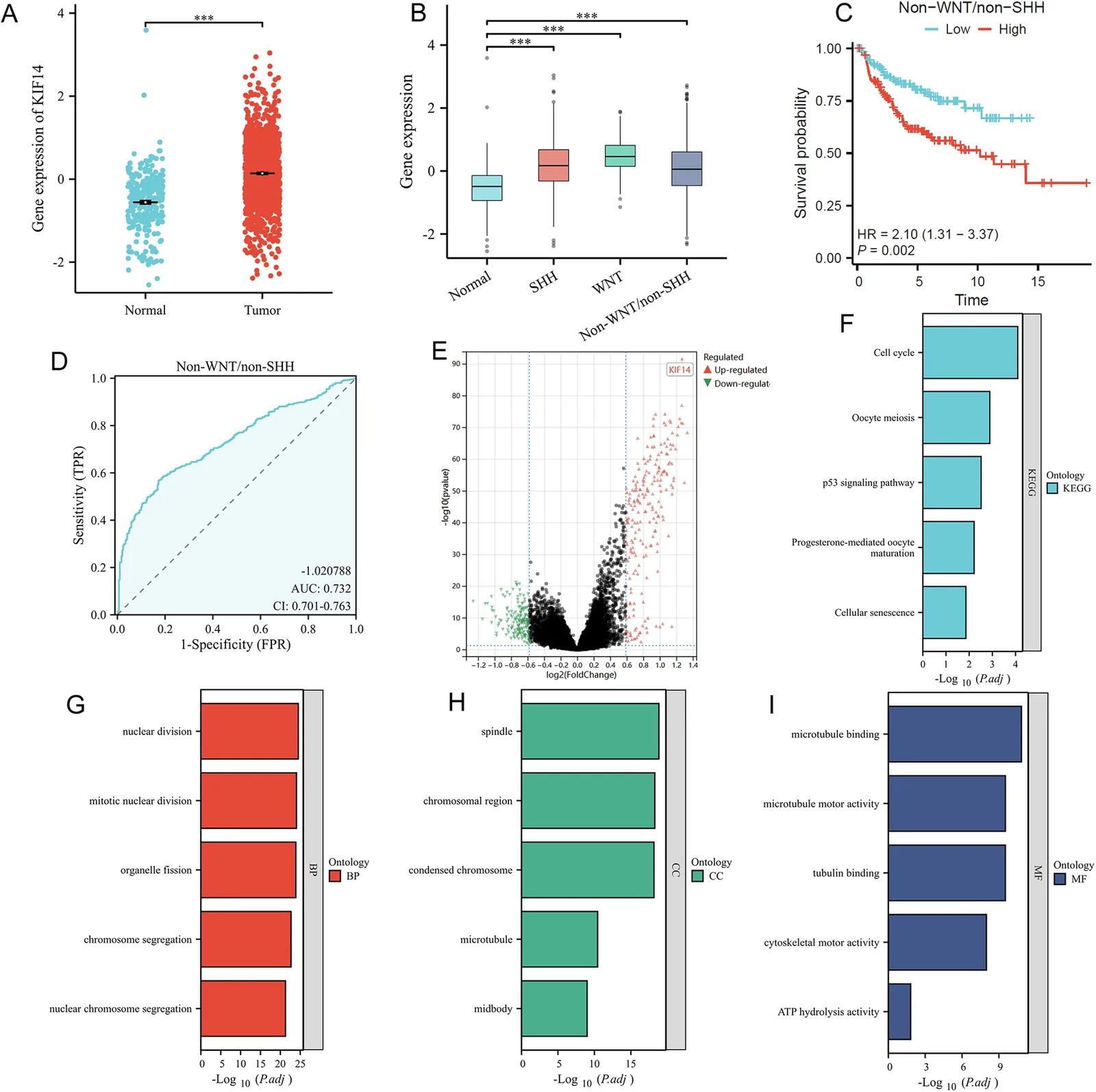
Prognostic and diagnostic value of KIF14 and its functional enrichment analysis in non-WNT/non-SHH MB. **A**, **B** The expression of KIF14 in different subtypes of MB and normal cerebellar tissues. **C** Kaplan–Meier survival curves of non-WNT/non-SHH MB patients were divided into high (red) or low (blue) groups according to the median value of KIF14 expression. **D** ROC curve analysis for KIF14 expression in non-WNT/non-SHH MB and normal cerebellar tissues. **E** Volcano plot showing the DEGs between non-WNT/non-SHH MB patients according to the median expression of KIF14. **F** Enrichment analysis of KEGG terms for KIF14-related DEGs. **G**–**I** Enrichment analysis of GO terms for KIF14-related DEGs. ^***^p < 0.001.

The mRNA expression levels of KIF14 were significantly elevated across all subtypes of MB (Fig. 1A, B). Kaplan–Meier survival analyses further revealed that higher KIF14 levels predicted unfavorable overall survival in the overall patient population, the SHH subgroup, and the non-WNT/non-SHH subgroup alike (Fig. S1A–C; Fig. 1C). Notably, this association was most pronounced in the non-WNT/non-SHH subgroup, in which the hazard ratio (HR) reached the highest level. To further evaluate its diagnostic value, we performed a Receiver operating characteristic (ROC) analysis using the KIF14 expression data from the GSE85217 dataset. ROC curve analysis was conducted to determine the diagnostic accuracy of KIF14 in distinguishing non-WNT/non-SHH medulloblastoma from normal tissues; the resulting area under the curve (AUC) of 0.732 (Fig. 1D) suggests moderate to good predictive ability. Together, these results suggest that KIF14 is elevated in MB and may serve as both a prognostic and diagnostic marker for non-WNT/non-SHH MB.

To further characterize the functional role of KIF14, we identified 9126 differentially expressed genes (DEGs) between the KIF14^high^ and KIF14^low^ non-WNT/non-SHH MB samples, comprising 5071 upregulated and 4055 downregulated genes (Fig. 1E). KEGG pathway enrichment analysis revealed that the DEGs were mainly involved the cell cycle, oocyte meiosis, and the p53 signaling pathway (Fig. 1F). Gene Ontology (GO) analysis further indicated that the DEGs were mainly enriched in terms related to nuclear division (GO-BP) (Fig. 1G), spindle components (GO-CC) (Fig. 1H), microtubule binding activity (GO-MF) (Fig. 1I).

### Oncogenic role of KIF14 in non-WNT/non-SHH MB

**Fig. 2:**
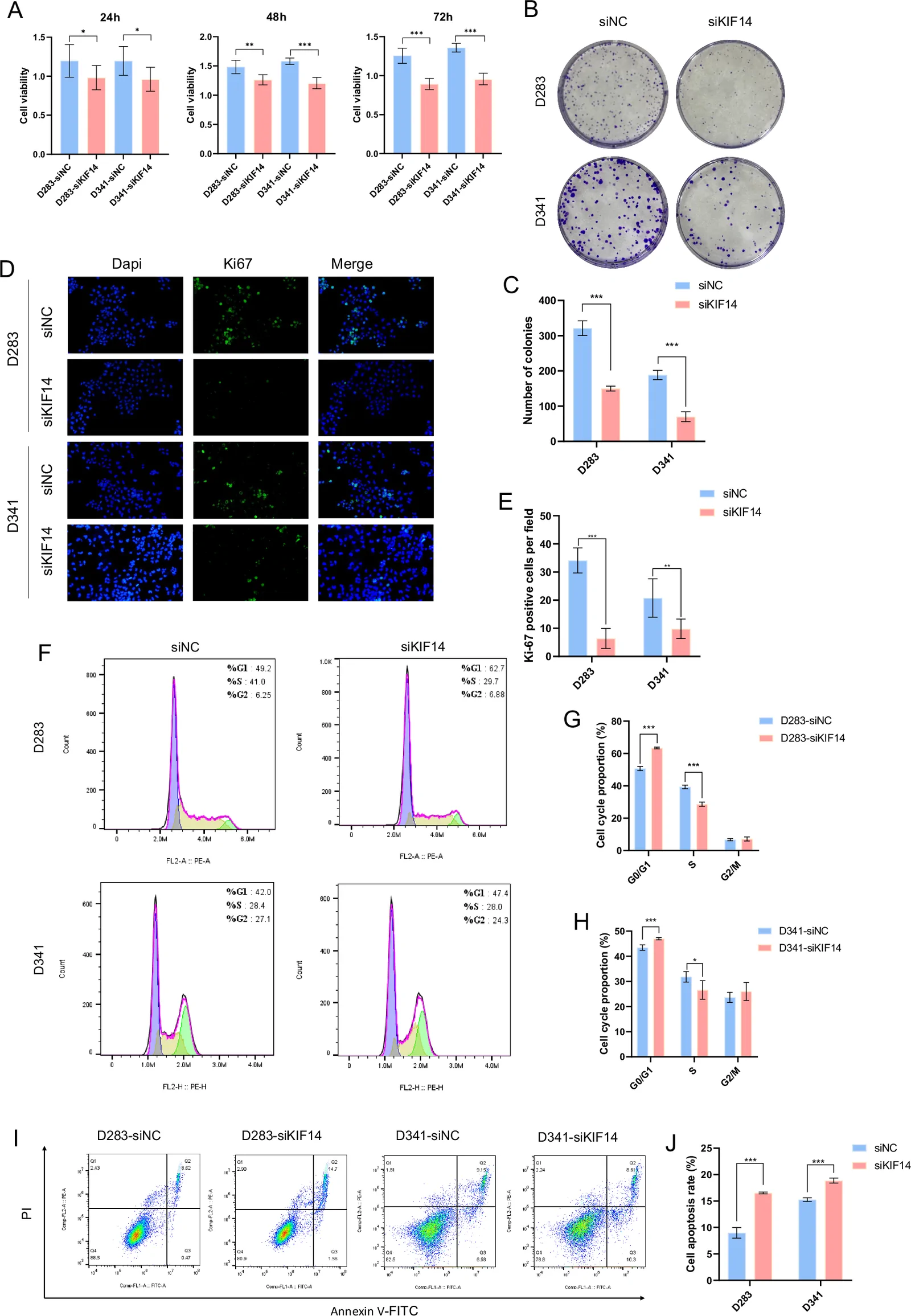
Oncogenic role of KIF14 in non-WNT/non-SHH MB. D283 and D341 cells were transfected with siNC or siKIF14. CCK-8 assay was used to evaluate the cell viability (*n* = 6) (**A**). Colony formation assays were used to evaluate the cell proliferation ability (*n* = 3) (**B**, **C**). Immunofluorescence staining showed the relative protein levels of Ki67 (*n* = 4) (**D**, **E**). Flow cytometry was used to evaluate cell cycle (*n* = 6) (**F**–**H**) and cell apoptosis (*n* = 5) (**I**, **J**). ^*^p < 0.05, ^**^p < 0.01, ^***^p < 0.001.

The knockdown efficiency of KIF14 was first verified by RT-qPCR and Western blot analyses following siRNA transfection. Among the designed siRNAs, siKIF14-1 exhibited the highest silencing efficiency and was thus selected for subsequent functional assays (Fig. S2A, B). The effects of KIF14 on the proliferation of non-WNT/non-SHH medulloblastoma cells were then assessed. CCK-8 assays demonstrated that KIF14 knockdown significantly decreased the viability of D283 and D341 cells at 24, 48, and 72 h post-seeding (Fig. 2A). Consistently, colony formation assays showed a marked decrease in colony-forming ability following KIF14 knockdown (Fig. 2B, C). Immunofluorescence analysis revealed reduced Ki67 expression, indicating a decline in proliferative activity upon KIF14 silencing (Fig. 2D, E). Next, we assessed the effects of KIF14 silencing on cell cycle and apoptosis by flow cytometry analysis. The results revealed that KIF14 silencing led to G0/G1 cell cycle arrest in both D283 and D341 cells (Fig. 2F–H). Moreover, the apoptotic rate was significantly increased in siKIF14-treated cells (Fig. 2I, J). Collectively, these findings demonstrate that KIF14 promotes the proliferation of non-WNT/non-SHH MB cells and that its silencing inhibits tumor growth by inducing G0/G1 cell cycle arrest and apoptosis.

### KIF14 silencing induces G0/G1 cell cycle arrest and apoptosis via activation of the p53 signaling pathway

**Fig. 3:**
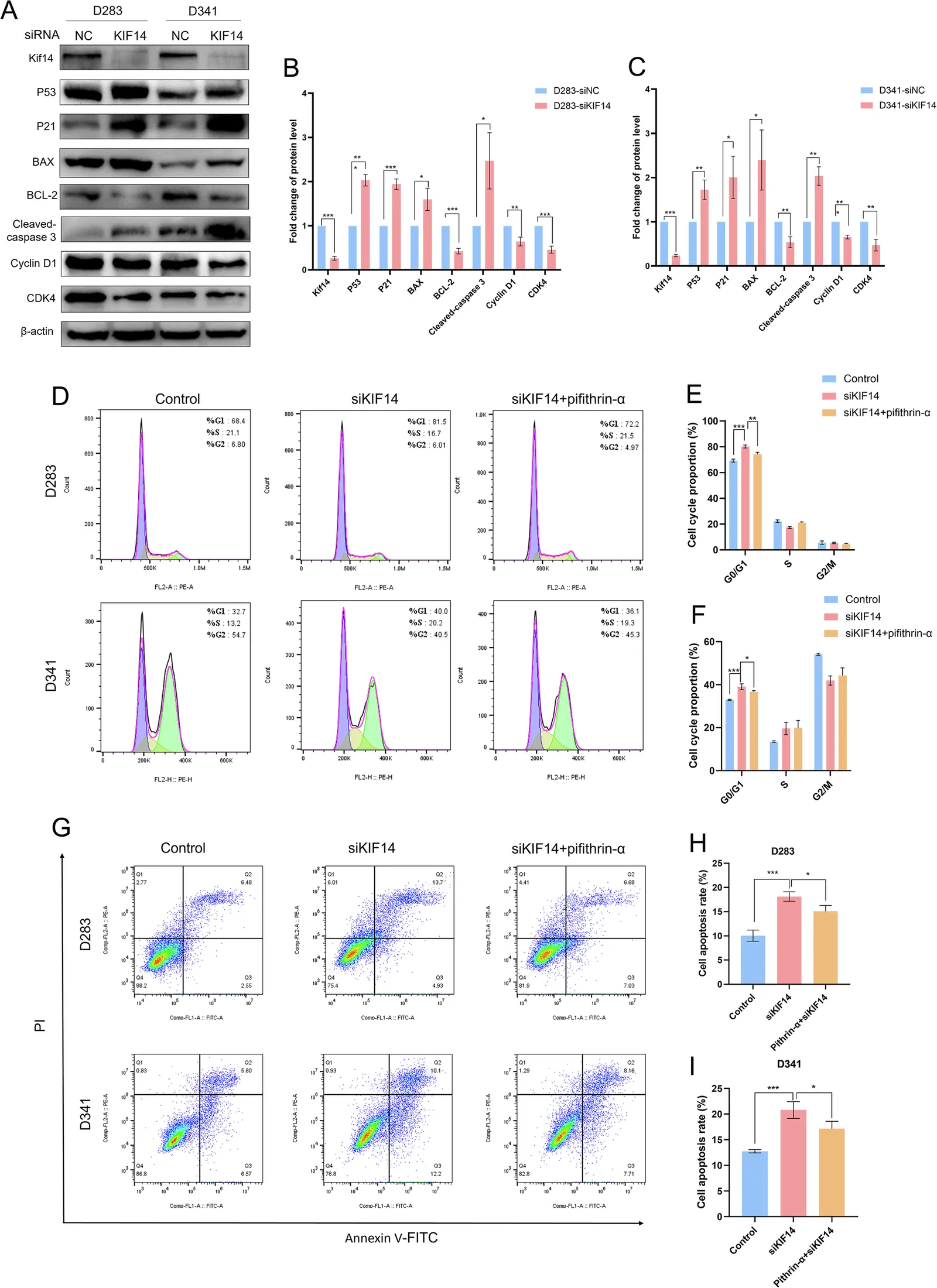
KIF14 silencing induces G0/G1 cell cycle arrest and apoptosis by regulating the p53 signaling pathway. Western blot showed that the protein levels of KIF14, p53, p21, Bax, Bcl-2, Cleaved-caspase 3, Cyclin D1, and CDK4 in D283 and D341 cells transfected with the indicated siRNA (*n* = 3) (**A**–**C**). Flow cytometry was used to detect the cell cycle distribution (*n* = 3) (**D**–**F**) and cell apoptosis (*n* = 3) (**G**–**I**) of D283 and D341 cells transfected with the indicated siRNA and treated with pifithrin-α (10 μl) for 24 h. ^*^p < 0.05, ^**^p < 0.01, ^***^p < 0.001.

Given that KEGG pathway analysis indicated enrichment of the p53 tumor suppressor pathway among KIF14-related DEGs (Fig. 1G), we investigated whether this pathway mediates the effects of KIF14 silencing on cell cycle progression and apoptosis. Western blotting analysis revealed that KIF14 knockdown increased the expression of p53 and its downstream effector p21 in non-WNT/non-SHH MB cells. In parallel, expression of the G0/G1 transition regulators Cyclin D1 and CDK4 was decreased, whereas the expression of pro-apoptotic proteins Bax and cleaved-caspase 3 was increased, but the levels of the pro-survival protein Bcl-2 were decreased (Fig. 3A–C). To determine whether the p53 signaling pathway mediates the anti-proliferative effects of KIF14 silencing, we employed PFT-α to inhibit p53 transcriptional activity. Our results showed that PFT-α treatment partially reversed the G0/G1 phase arrest and apoptosis triggered by KIF14 knockdown (Fig. 3D–I). These results suggest that KIF14 silencing activates the p53 signaling pathway, which in turn contributes to cell cycle arrest and apoptosis in non-WNT/non-SHH MB cells.

### KIF14 silencing activates the p53 signaling pathway by regulating nucleocytoplasmic shuttling of 53BP1

**Fig. 4:**
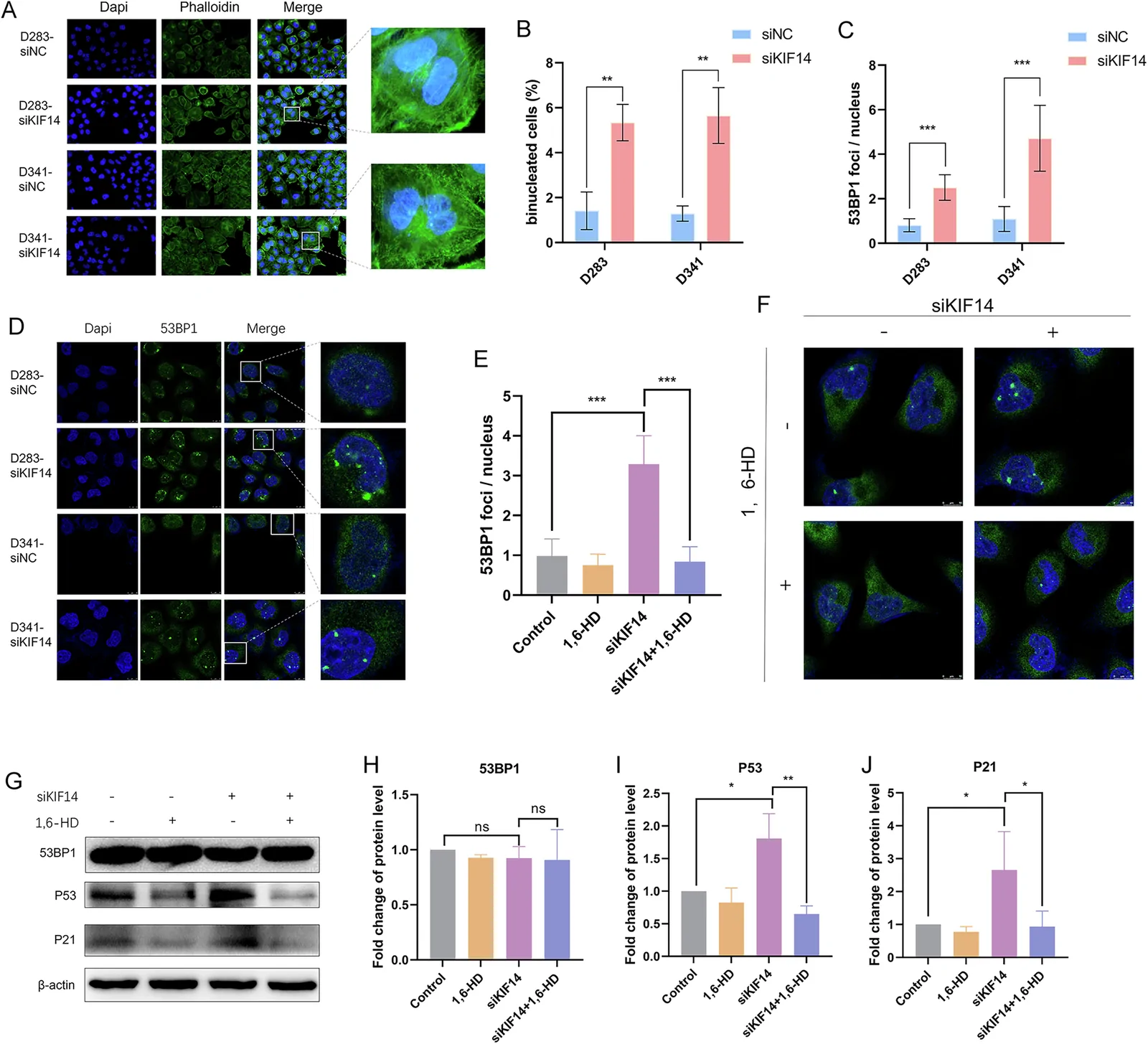
KIF14 silencing activates the P53 signaling pathway by regulating nucleocytoplasmic shuttling of 53BP1. Immunofluorescence showed that D283 and D341 cells transfected with the indicated siRNA were stained with DAPI and anti-phalloidin antibody (**A**). Quantification of binucleated D283 and D341 cells in the indicated transfections (*n* = 3) (**B**). Immunofluorescence staining showed 53BP1 foci per nucleus in D283 and D341 cells transfected with the indicated siRNA and treated with 1,6-HD (*n* = 4) (**C**–**F**). Western blot showed that the protein levels of 53BP1, p53, and p21 in D341 cells transfected with the indicated siRNA and treated with 1,6-HD (*n* = 3) (**G**–**J**). ^*^p < 0.05, ^**^p < 0.01, ^***^p < 0.001.

To elucidate the mechanism by which KIF14 silencing activates the p53 pathway in non-WNT/non-SHH MB cells, we investigated potential downstream factors of KIF14 responsible for p53 activation. Given the established role of KIF14 in intercellular bridge cleavage during mitosis, we assessed the effect of KIF14 silencing on cytokinesis. Silencing KIF14 resulted in delayed cytokinesis, as evidenced by a significant increase in binucleated cells (Fig. 4A, B). Previous studies have shown that 53BP1, a p53-binding protein, is essential for p53 activation during mitotic duration prolongation caused by centrosome loss [18]. Therefore, we further examined whether 53BP1 mediates the activation of the p53 signaling pathway in the delayed cytokinesis process induced by KIF14 silencing. Immunofluorescence analysis revealed that KIF14 silencing led to increased accumulation of nuclear 53BP1 foci (Fig. 4C, D), without altering total 53BP1 protein levels (Fig. 4G, H). To further probe the role of 53BP1 foci in p53 activation, we used 1,6-hexanediol (1,6-HD), a phase separation inhibitor that disrupts the formation of 53BP1 nuclear foci (Fig. 4E, F). Treatment with 1,6-HD blocked the accumulation of 53BP1 foci and significantly reduced the upregulation of p53 and p21 induced by KIF14 silencing (Fig. 4G–J). These findings suggest that KIF14 silencing promotes the accumulation of 53BP1 nuclear foci, which in turn mediates activation of the p53 signaling pathway in non-WNT/non-SHH MB cells. Notably, this regulation occurs without changes in the overall expression level of the 53BP1 protein.

### KIF14 silencing sensitizes non-WNT/non-SHH MB cells to cisplatin chemotherapy in vitro by impairing homologous recombination (HR)

**Fig. 5:**
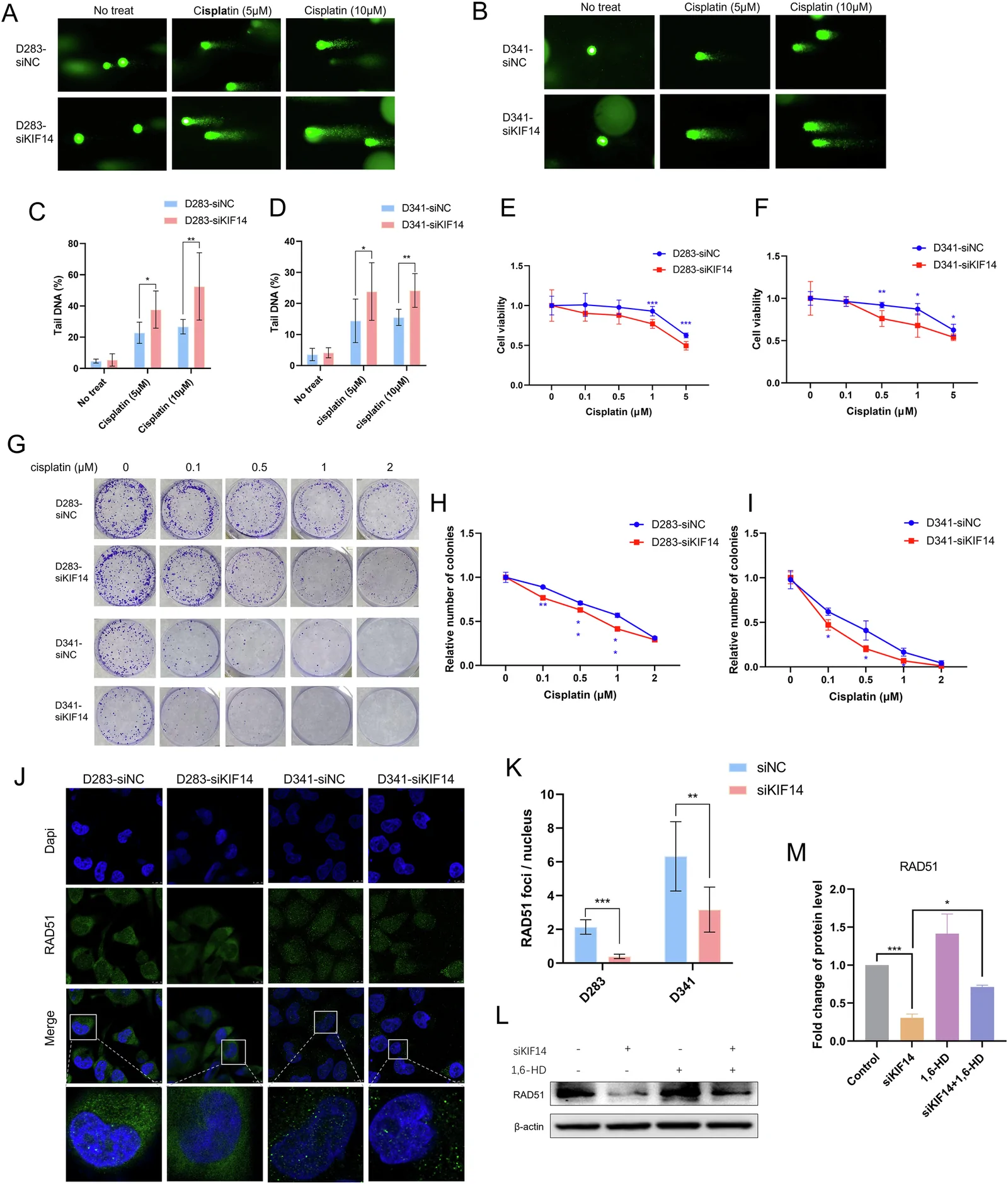
KIF14 silencing sensitizes cisplatin chemotherapy in vitro by impairing homologous recombination. Alkaline comet assay was used to detect DNA damage in D283 and D341 cells transfected with siRNA and treated with cisplatin (**A**, **B**). Tail DNA percentages of cells were examined in each treatment group (*n* = 4) (**C**, **D**). CCK-8 assay was used to detect the viability of D283 and D341 cells transfected with siRNA and treated with cisplatin (*n* = 6) (**E**, **F**). Colony formation assays were used to evaluate the proliferation of D283 and D341 cells transfected with siRNA and treated with cisplatin (*n* = 3) (**G**–**I**). Immunofluorescence staining showed RAD51 foci per nucleus in D283 and D341 cells transfected with the indicated siRNA (*n* = 3) (**J**, **K**). Western blot showed that the protein level of RAD51 in D341 cells transfected with the indicated siRNA and treated with 1,6-HD (*n* = 3) (**L**, **M**). ^*^p < 0.05, ^**^p < 0.01, ^***^p < 0.001.

53BP1 is a critical protein involved in the repair of DNA double-strand breaks (DSBs), which plays a significant role in the effectiveness of DNA-damaging chemotherapeutic agents. To assess the impact of KIF14 silencing on cisplatin-induced DNA damage, we performed a neutral alkaline comet assay, which revealed that knockdown of KIF14 increased the percentage of tail DNA in D283 and D341 cells treated with cisplatin. This result indicates that KIF14 silencing enhances the DSBs caused by cisplatin on non-WNT/non-SHH MB cells (Fig. 5A–D). Moreover, CCK-8 assays showed that KIF14 silencing reduced the cell viability of non-WNT/non-SHH MB cells following cisplatin treatment (Fig. 5E, F). Colony formation assays also showed that after 10 days of cisplatin treatment, the number of colonies formed in the KIF14 silencing group was significantly lower than that in the control group, suggesting enhanced sensitivity to cisplatin (Fig. 5G–I). RAD51 is a central protein involved in HR. RAD51 competes with 53BP1 for binding to DSBs in the nucleus. Immunofluorescence analysis showed a decrease in the frequency of RAD51 nuclear foci in KIF14-silenced non-WNT/non-SHH MB cells (Fig. 5J, K). Notably, inhibiting 53BP1 foci formation with 1,6-HD partially restored RAD51 protein levels (Fig. 5L, M). Overall, these data suggest that KIF14 silencing sensitizes non-WNT/non-SHH MB cells to cisplatin treatment by impairing HR repair mechanisms, as evidenced by the reduced accumulation of RAD51 nuclear foci and enhanced DNA damage response.

### KIF14 silencing sensitizes non-WNT/non-SHH MB to cisplatin, effectively inhibiting tumor growth in the intracranial orthotopic tumor model

**Fig. 6:**
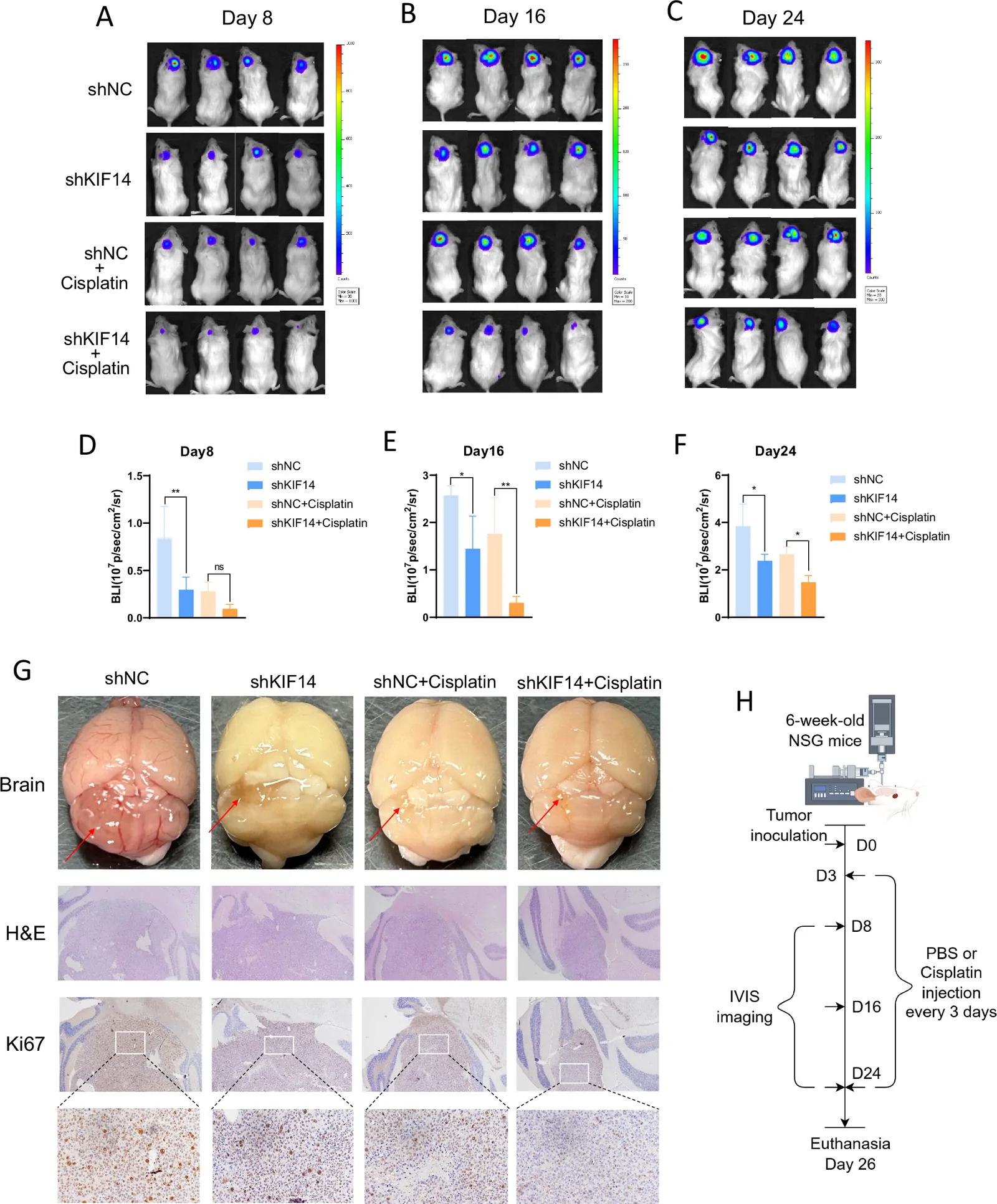
KIF14 silencing sensitizes cisplatin, which effectively inhibits the growth of MB in the intracranial orthotopic tumor model. Bioluminescence images of mice from each treatment group on the 8th, 16th, and 24th days after implantation were shown (*n* = 4) (**A**–**F**). The gross appearance of the whole brain of mice in each treatment group was shown. H&E staining and Ki67 immunochemistry of the mouse brain showed the tumor and proliferating cells in each treatment group (**G**). The experimental timeline for the intracranial orthotopic xenograft assay is depicted schematically (**H**). ^*^p < 0.05, ^**^p < 0.01.

To assess whether KIF14 silencing enhances the sensitivity of non-WNT/non-SHH MB cells to cisplatin and inhibits tumor growth in vivo, we established orthotopic tumors in NSG mice using D341 cells stably expressing luciferase, either with shNC or shKIF14. After cell implantation, cisplatin (2 mg/kg) was administered intraperitoneally every 3 days for 24 days, and tumor growth was monitored using IVIS imaging (Fig. 6H). IVIS imaging of these mice at days 8, 16, and 24 after implantation revealed that shKIF14 alone significantly inhibited tumor growth, while the combination of shKIF14 and cisplatin further inhibited tumor growth (Fig. 6A–F). Upon sacrifice of the mice and collection of brain tissue, gross inspection and H&E staining confirmed the presence of tumor tissues in the cerebellar region of each group. Further, there was a significant decrease in the proliferation marker Ki67–positive cells but a significant increase in cleaved-caspase 3 positive cells in the combination treatment of shKIF14 and cisplatin compared with the control and cisplatin-alone groups. (Fig. 6G; Fig. S3). Taken together, these results showed that KIF14 silencing sensitizes non-WNT/non-SHH MB to cisplatin treatment, effectively inhibiting tumor growth in vivo.

## Discussion

**Fig. 7:**
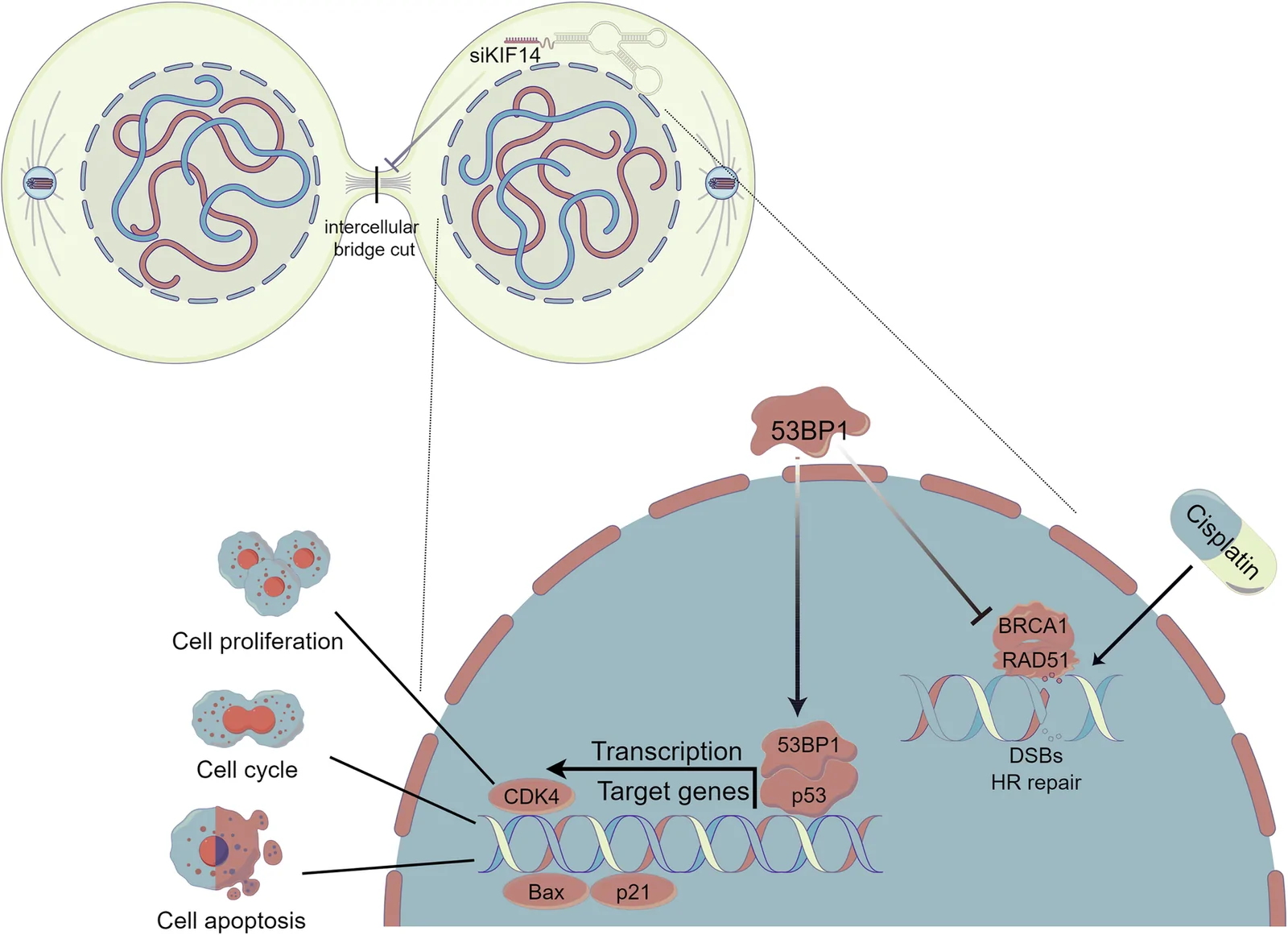
A schematic diagram showing KIF14 silencing activating the p53 signaling pathway and enhancing cisplatin sensitivity through the 53BP1 cytoplasmic shuttle in non-WNT/non-SHH MB.

In this study, we demonstrated that KIF14 is highly expressed in non-WNT/non-SHH MB and is associated with poor patient prognosis. Silencing KIF14 induces cell cycle arrest and apoptosis through the p53 signaling pathway, while also enhancing the DNA damage response to cisplatin, thereby exhibiting antitumor effects. Furthermore, KIF14 silencing activates the p53 signaling pathway and impairs DNA HR repair by regulating the nucleocytoplasmic shuttling of 53BP1 (Fig. 7).

The non-WNT/non-SHH MB category is characterized by considerable clinical and genetic heterogeneity. Unlike WNT and SHH subgroups, patients with non-WNT/non-SHH MB are more likely to lack identifiable somatic driver mutations [6, 19]. This means that the research strategies focused on targeting specific mutated genes may not be directly applicable. Evidence indicates that MB cells share molecular profiles with granular neuron progenitors, and MCPH genes are required for the proliferation of progenitor cells [20, 21]. One such gene, KIF14, is commonly mutated in MCPH20 syndrome. KIF14 is highly expressed in the placenta and fetal brain tissues, while it is expressed at low levels in postnatal brain tissues [16]. In addition, KIF14 is an important protein that regulates microtubule dynamics during mitosis, which is of great significance for the malignant proliferation of tumors [22]. KIF14 possesses an internal motor domain with a high affinity for microtubules and helps protect microtubules from depolymerization [23]. KIF14 silencing leads to difficulty in the cleavage of intracellular bridges during anaphase of mitosis [22]. This is consistent with our results, whereas KIF14 knockdown caused an increase in binucleate cells in non-WNT/non-SHH MB, suggesting a delay in cytokinesis.

To further investigate the role of KIF14 in non-WNT/non-SHH MB, we first conducted a bioinformatics analysis. The enrichment results from GO and KEGG pathway analyses suggest that KIF14 is mainly involved in the biological processes of mitosis and is closely related to the cell cycle and the p53 signaling pathway. As both a tumor suppressor and transcription factor, p53 plays an important role in mediating cell cycle arrest and triggering apoptosis [24]. Our results demonstrated that KIF14 silencing resulted in increased p53 expression, cell cycle arrest in the G0/G1 phase, and increased apoptosis in non-WNT/non-SHH MB cells. P53 influences the transcription of pro-apoptotic protein Bax, disrupting the balance between Bax and Bcl-2, leading to cell apoptosis [24]. These findings suggest that p53 may mediate the pro-apoptotic effect of KIF14 silencing on non-WNT/non-SHH MB cells. Regarding cell cycle regulation, p21, an important effector molecule downstream of p53, functions as an inhibitor of cyclin-dependent kinases (CDKs). CDK4 binds to cyclin D1 to facilitate the transition from the G0/G1 phase to the S phase [25, 26]. In our study, KIF14 silencing results in increased p21 expression and decreased CDK4 and cyclin D1 expression in non-WNT/non-SHH MB cells, causing G0/G1 phase cell cycle arrest. The pro-apoptotic and cell cycle arrest effects induced by KIF14 silencing were partially reversed by treatment with a p53 inhibitor. These results suggest that the p53 signaling pathway mediates the pro-apoptotic and cell cycle arrest effects of KIF14 silencing in non-WNT/non-SHH MB.

In normal neural progenitor cells, genotoxic stress caused by delayed mitosis can also trigger p53-dependent apoptosis [27]. However, the mechanisms that lead to p53 activation are not fully understood. As a p53-binding protein, 53bp1 participates in the activation of p53 after nuclear translocation during the anaphase of mitosis. Studies have shown that 53BP1 disassociates from centromeres in a time-dependent manner and forms nuclear foci in response to mitotic delay induced by centrosome loss [28]. In this study, we observed that the expression level of the 53BP1 protein did not significantly change following KIF14 silencing and cytokinesis delay. However, there was a marked increase in the accumulation of 53BP1 nuclear foci. The upregulation of p53 and p21 expression induced by silencing KIF14 in non-WNT/non-SHH MB cells was reversed when the phase separation inhibitor 1,6-HD was used to block the formation of 53BP1 nuclear foci. These findings are consistent with the concept of the mitotic surveillance pathway (MSP), in which the formation of 53BP1 nuclear foci provides a platform for complex formation with p53, enhancing its ability to transactivate target genes [29].

Cisplatin remains a cornerstone in MB therapy, primarily through its interaction with bases on the DNA strand, resulting in the formation of interstrand crosslinks and DNA DSBs [30, 31]. In addition to its role in p53-mediated tumor suppressor effects, 53BP1 also plays a crucial role in DNA DSBs repair [29]. HR DNA repair, which occurs mainly during the S and G2 phases of the cell cycle, is essential for maintaining genomic stability [32, 33]. As a key protein in HR repair, the loading of RDA51 onto 3′ single-stranded DNA depends on the resection of the 5′-3′ end of BRCA1. 53BP1 interacts with PTIP and RIF1/Shieldin effector complexes in a phosphorylation-dependent manner to antagonize the key steps of 5′-3′ end resection and RAD51 loading coordinated by BRCA1 [34, 35]. Our results demonstrated that KIF14 silencing led to the accumulation of 53BP1 nuclear foci, accompanied by a reduction in both RAD51 expression and the number of RAD51 nuclear foci. Notably, the phase separation inhibitor of 53BP1 was able to partially reverse the downregulation of RAD51 expression. The comet assay results further indicated that KIF14 silencing exacerbated cisplatin-induced DNA DSBs in non-WNT/non-SHH MB cells. In the assay of cell proliferation ability, KIF14 silencing enhanced the inhibitory effects of cisplatin on colony formation and cell viability. Moreover, in the xenograft mouse model, the same dose of cisplatin administered to shKIF14-injected MB cells resulted in a significant inhibition in orthotopic tumor growth.

In conclusion, this study highlights the elevated expression of KIF14 in non-WNT/non-SHH MB and its association with poor patient prognosis. We have provided new insights into the role of KIF14 in tumor pathogenesis. Our findings demonstrate that KIF14 silencing inhibits tumor growth and enhances cisplatin sensitivity by regulating the nuclear targeting of 53BP1. These results suggest that targeting KIF14 could be a promising strategy to improve cisplatin efficacy and potentially offer more effective treatment options for patients with the genetically heterogeneous non-WNT/non-SHH MB subgroup. This study could lay the groundwork for the development of innovative therapies aimed at increasing cisplatin sensitivity and improving patient outcomes in this challenging cancer subtype.

## Materials and methods

### Bioinformatics analysis

Datasets GSE124814 (including the transcriptome sequencing data of 1350 MB and 291 normal cerebellum samples) and GSE85217 (including transcriptome sequencing data of 763 MB samples and matched clinical information) were retrieved from the Gene Expression Omnibus (GEO) database (https://www.ncbi.nlm.nih.gov/geo/). Box plots and scatter plots were used to show the transcriptome levels of KIF14 in MB samples and normal cerebellar tissues. According to the median expression level of KIF14, the RNA-seq expression matrix was divided into the KIF14^high^ expression group and the KIF14^low^ expression group. The Kaplan–Meier plotter was used to estimate the correlation between KIF14 expression and the overall survival of patients with MB, and the log-rank *p*-value of the 95% confidence interval and hazard ratio (HR) were calculated. ROC analysis was used to evaluate the diagnostic and predictive ability of KIF14, and the area under the curve (AUC) > 0.7 indicated satisfactory predictive ability. GO and KEGG analyses of differential expression genes (DEGs) were performed using the R package clusterProfiler.

### Cell culture and transfection

The human non-WNT/non-SHH MB cell lines (D283 and D341) were obtained from ATCC [36]. D283 and D341 cell lines were cultured in Dulbecco’s modified Eagle’s medium (DMEM, Biological Industries, Israel) supplemented with 10% fetal bovine serum (FBS, Gibco, USA) and 1% penicillin-streptomycin (Genom, China) and were incubated in a humidified incubator with 5% CO_2_ at 37 °C. We transfected the siRNA (sequences summarized in Table S1) with Lipofectamine 3000 (Invitrogen, USA) according to the manufacturer’s recommended instructions.

### Generation of stable cell lines

The cells were transduced with the LV16 (U6/Luciferase17&Puro) lentivirus RNAi expression system (Suzhou GenePharma, China) expressing shRNA-KIF14 (sequences summarized in Table S1). The multiplicity of infection (MOI) was 20. The virus was transfected with serum-free medium for 12 h at 37 °C. The medium was changed and replaced with complete medium, and subsequent experiments were carried out after culturing for 48 h at 37 °C. Subsequently, cells were cultured in complete medium instead, and puromycin was added to screen stable transplants for 48 h.

### RT-qPCR

Total RNA was extracted from freshly harvested cultured cells using TRIzol reagent (Invitrogen, Thermo Fisher Scientific, USA). RNA purity and concentration were determined with a NanoDrop spectrophotometer (ND-1000, Thermo Fisher Scientific, USA). Subsequently, 1 μg of total RNA was reverse-transcribed into cDNA using a commercial Reverse Transcription kit (Promega Corporation, USA). Quantitative PCR amplification was performed with SYBR Green PCR Master Mix (Toyobo Life Science, Japan) on an ABI 7500 Real-time PCR System (Thermo Fisher Scientific, USA). The primer sequences used in this study are listed in Table S2. Relative gene expression levels were calculated using the 2^−ΔΔCq^ method.

### Cell proliferation assay

D283 and D341 cells were seeded into 96-well plates at 1 × 10^3^/well. 10 µL of CCK-8 solution was added to each well and used to test cell viability. The absorbance was recorded at 450 nm using a microplate reader (Thermo Fisher Scientific, China). For the colony-forming assay, A total of 500 cells per well were cultivated in 6-well plates for 10 days at 37 °C. After medium removal, the cells were fixed and stained with 0.1% crystal violet (Solarbio, China). Finally, the mean number of visible colonies was counted.

### Cell cycle and apoptosis were analyzed by flow cytometry

Cells from different groups were digested with trypsin and harvested by centrifugation. For cell cycle detection, the cells were fixed with 70% alcohol at 4 °C overnight and stained with RNase A-containing PI buffer for 30 min. The cell cycle was detected by a flow cytometer (BD Biosciences, USA). For apoptosis detection, cells were stained with annexin V and PI, following the manufacturer’s instructions (beyotime, China). Cell apoptosis was detected by a flow cytometer. FlowJo software was used for data analysis.

### Protein extraction and Western blotting (WB)

Proteins from cells were extracted using RIPA lysis buffer (beyotime, China) supplemented with PMSF (Thermo Scientific, USA), and the protein concentration was detected by the BCA method (ab102536; Abcam, USA). Then the protein lysates were separated by 8–12% sodium dodecyl sulfate–polyacrylamide gel electrophoresis and transferred to a polyvinylidene difluoride membrane. Primary antibodies against KIF14 (DF15723; Affinity Biosciences, USA), p53 (AF0879; Affinity Biosciences, USA), p21 (AF6290; Affinity Biosciences, USA), Bax (AF0120; Affinity Biosciences, USA), Bcl-2 (AF6139; Affinity Biosciences, USA), Cleaved-Caspase 3 (Asp175) (AF7022; Affinity Biosciences, USA), Cyclin D1 (AF0931; Affinity Biosciences, USA), CDK4 (DF6102; Affinity Biosciences, USA), 53BP1 (ab175933, Abcam, USA), RAD51 (ab133534, Abcam, USA), and β-actin (AF7018; Affinity Biosciences, USA) were used. Horseradish peroxidase-coupling secondary antibody (S0001, Affinity Biosciences, USA) was used. At last, the membrane was detected using the Bio-Rad Molecular Imager FX.

### Immunofluorescence and imaging

The cells were fixed at RT with 4% PFA for 15 min, and permeabilized in 0.2% Triton X-100 for 10 min. After blocking in 10% goat serum for 45 min, the cells were incubated with primary antibody at RT for 2 h. Then the cells were incubated with AlexaFluor 488 (goat anti-rabbit IgG, Abcam, USA) for 1 h at RT, stained with 0.5 μg/ml DAPI for 10 min. Finally, the Leica TCS SP5 confocal system (Leica Microsystems, Germany) was used to collect images.

### Comet assay

According to the manufacturer’s instructions, the Comet Assay kit (4250-050-K, Trevigen, USA) was used to analyze DNA DSBs in each group of cells. Images were taken using an inverted microscope (Olympus Corporation, Japan). The percentage of tail DNA was quantified in cells for each condition using the CASP software.

### Intracranial orthotopic model

Luciferase-expressing LV-shKIF14 or LV-shNC D341 cells (1 × 10^5^ cells per mouse in 5 μl total) were injected into the cerebellum (2 mm posterior to the lambda suture, 2 mm lateral to the midline, and 2 mm deep) of 6-week-old NSG mice using a Hamilton neurosyringe connected to a stereotactic apparatus (David Kopf Instruments). Mice were randomly divided into four groups: shNC, shKIF14, shNC + cisplatin (2 mg/kg), and shKIF14 + cisplatin (2 mg/kg), and treatments were given intraperitoneally every three days for 24 days. Mice were imaged on the IVIS Lumina Imaging System (Xenogen, USA) on the 8th, 16th, and 24th days after cell injection. Mice were sacrificed on day 26, and their brains were harvested, and tumor tissues were photographed and embedded in paraffin. H&E staining and immunohistochemical analyses for Ki67 and cleaved-caspase-3 were performed on tumor tissue sections to confirm the histological features and to evaluate cellular proliferation and apoptosis.

### Statistical analysis

All statistical analyses were performed with GraphPad Prism5 software. Data were shown as mean ± SD of at least three independent experiments. Two-tailed unpaired Student’s *t*-test, Kaplan‒Meier analysis, and one-way analysis of variance (ANOVA) were applied appropriately for statistical comparisons in this study. *p* < 0.05 was considered statistically significant.

## Supplementary information

WB raw data

Supplementary Material

## Data Availability

All data generated or analyzed during this study are available from the corresponding author upon reasonable request.

## References

[1] Ostrom Q, Patil N, Cioffi G, Waite K, Kruchko C, Barnholtz-Sloan J. CBTRUS statistical report: primary brain and other central nervous system tumors diagnosed in the United States in 2013-2017. Neuro-Oncol. 2020;22:iv1–iv96. 10.1093/neuonc/noaa200 PMC7596247 33123732

[2] Cavalli F, Remke M, Rampasek L, Peacock J, Shih D, Luu B, et al. Intertumoral heterogeneity within medulloblastoma subgroups. Cancer cell. 2017;31:737–54.e6. 10.1016/j.ccell.2017.05.005 PMC6163053 28609654

[3] Ramaswamy V, Remke M, Bouffet E, Bailey S, Clifford S, Doz F, et al. Risk stratification of childhood medulloblastoma in the molecular era: the current consensus. Acta Neuropathol. 2016;131:821–31. 10.1007/s00401-016-1569-6 PMC4867119 27040285

[4] Northcott P, Jones D, Kool M, Robinson G, Gilbertson R, Cho Y, et al. Medulloblastomics: the end of the beginning. Nat Rev Cancer. 2012;12:818–34. 10.1038/nrc3410 PMC3889646 23175120

[5] Louis D, Perry A, Wesseling P, Brat D, Cree I, Figarella-Branger D, et al. The 2021 WHO Classification of Tumors of the Central Nervous System: a summary. Neuro-Oncol. 2021;23:1231–51. 10.1093/neuonc/noab106 PMC8328013 34185076

[6] Northcott P, Buchhalter I, Morrissy A, Hovestadt V, Weischenfeldt J, Ehrenberger T, et al. The whole-genome landscape of medulloblastoma subtypes. Nature. 2017;547:311–7. 10.1038/nature22973 PMC5905700 28726821

[7] Eric MT, David A, Daniel L. Current medulloblastoma subgroup specific clinical trials. Transl Pediatr. 2020;9:157-62. 10.21037/tp.2020.03.03 PMC7237968 32477916

[8] Catena K, Lea H, Melanie S, Tara L, Carolin G, Sina N, et al. Intraventricular SHH inhibition proves efficient in SHH medulloblastoma mouse model and prevents systemic side effects. Neuro Oncol. 2023;26:609-22. 10.1093/neuonc/noad191 PMC10995518 37767814

[9] Gibson P, Tong Y, Robinson G, Thompson MC, Currle DS, Eden C, et al. Subtypes of medulloblastoma have distinct developmental origins. Nature. 2010;468:1095–9. 10.1038/nature09587 PMC3059767 21150899

[10] Ryan S ON, Todd A S, Nasser MR. Same but different: pleiotropy in centrosome-related microcephaly. Mol Biol Cell. 2018;29(3):241-246. 10.1091/mbc.E17-03-0192 PMC5996963 29382806

[11] Zaqout S, Morris-Rosendahl D, Kaindl A. Autosomal recessive primary microcephaly (MCPH): an update. Neuropediatrics. 2017;48:135–42. 10.1055/s-0037-1601448 28399591

[12] O’Neill R, Schoborg T, Rusan N. Same but different: pleiotropy in centrosome-related microcephaly. Mol Biol Cell. 2018;29:241–6. 10.1091/mbc.E17-03-0192 PMC5996963 29382806

[13] Iegiani G, Di Cunto F, Pallavicini G. Inhibiting microcephaly genes as alternative to microtubule targeting agents to treat brain tumors. Cell Death Dis. 2021;12:956. 10.1038/s41419-021-04259-6 PMC8523548 34663805

[14] Madeline Louise R, Marijn FS, Virginie M, Cecile J, Marine A, Mohammadjavad P, et al. Loss-of-function mutations in KIF14 cause severe microcephaly and kidney development defects in humans and zebrafish. Hum Mol Genet. 2018;28:778-95. 10.1093/hmg/ddy381 PMC6381319 30388224

[15] Michael C, Mao M, Matthew B, Paul W, Sammy K, Carolyn B, et al. RNA interference-mediated silencing of mitotic kinesin KIF14 disrupts cell cycle progression and induces cytokinesis failure. Mol Cell Biol. 2006;26:3853-63. 10.1128/MCB.26.10.3853-3863.2006 PMC1488988 16648480

[16] Timothy WC, Annie H, Ming-Sound T, Brenda LG. KIF14 is a candidate oncogene in the 1q minimal region of genomic gain in multiple cancers. Oncogene. 2005;24:4741-53. 10.1038/sj.onc.1208641 15897902

[17] Kay Ka-Wai L, Yan Q, Tian X, Aden Ka-Yin C, Zhen-Yu Z, Abudumijiti A, et al. The kinesin KIF14 is overexpressed in medulloblastoma and downregulation of KIF14 suppressed tumor proliferation and induced apoptosis. Lab Invest. 2017;97:946–61. 10.1038/labinvest.2017.48 28504687

[18] Thao PP, Aubrey LM, Christina AB, Junsu L, Alisa A, Andrea T, et al. Centrosome defects cause microcephaly by activating the 53BP1-USP28-TP53 mitotic surveillance pathway. EMBO J. 2020;40:e106118. 10.15252/embj.2020106118 PMC7780150 33226141

[19] David RG, Gudrun F, Till M, Kristian WP. The current landscape of targeted clinical trials in non-WNT/non-SHH medulloblastoma. Cancers. 2022;14:679. 10.3390/cancers14030679 PMC8833659 35158947

[20] Zhang L, He X, Liu X, Zhang F, Huang L, Potter A, et al. Single-cell transcriptomics in medulloblastoma reveals tumor-initiating progenitors and oncogenic cascades during tumorigenesis and relapse. Cancer Cell. 2019;36:302–18.e7. 10.1016/j.ccell.2019.07.009 PMC6760242 31474569

[21] Vladoiu M, El-Hamamy I, Donovan L, Farooq H, Holgado B, Sundaravadanam Y, et al. Childhood cerebellar tumours mirror conserved fetal transcriptional programs. Nature. 2019;572:67–73. 10.1038/s41586-019-1158-7 PMC6675628 31043743

[22] Arora K, Talje L, Asenjo A, Andersen P, Atchia K, Joshi M, et al. KIF14 binds tightly to microtubules and adopts a rigor-like conformation. J Mol Biol. 2014;426:2997–3015. 10.1016/j.jmb.2014.05.030 24949858

[23] Gruneberg U, Neef R, Li X, Chan E, Chalamalasetty R, Nigg E, et al. KIF14 and citron kinase act together to promote efficient cytokinesis. J Cell Biol. 2006;172:363–72. 10.1083/jcb.200511061 PMC2063646 16431929

[24] Hu J, Cao J, Topatana W, Juengpanich S, Li S, Zhang B, et al. Targeting mutant p53 for cancer therapy: direct and indirect strategies. J Hematol Oncol. 2021;14:157. 10.1186/s13045-021-01169-0 PMC8480024 34583722

[25] Fischer M, Schade A, Branigan T, Müller G, DeCaprio J. Coordinating gene expression during the cell cycle. Trends Biochem Sci. 2022;47:1009–22. 10.1016/j.tibs.2022.06.007 35835684

[26] Swaffer M, Jones A, Flynn H, Snijders A, Nurse P. CDK substrate phosphorylation and ordering the cell cycle. Cell. 2016;167:1750–61.e16. 10.1016/j.cell.2016.11.034 PMC5161751 27984725

[27] Swaffer M, Jones A, Flynn H, Snijders A, Nurse P. CDK substrate phosphorylation and ordering the cell cycle. Cell. 2016;167(7):1750-1761. 10.1016/j.cell.2016.11.034 PMC5161751 27984725

[28] Louis-Jan P, John JM, Emily EM, Ashley LL, Aussie S, Edward S, et al. Prolonged mitosis of neural progenitors alters cell fate in the developing brain. Neuron. 2016;89:83-99. 10.1016/j.neuron.2015.12.007 PMC4706996 26748089

[29] Burigotto M, Vigorito V, Gliech C, Mattivi A, Ghetti S, Bisio A, et al. PLK1 promotes the mitotic surveillance pathway by controlling cytosolic 53BP1 availability. EMBO Rep. 2023;24:e57234. 10.15252/embr.202357234 PMC10702821 37888778

[30] Nakata S, Murai J, Okada M, Takahashi H, Findlay T, Malebranche K, et al. Epigenetic upregulation of Schlafen11 renders WNT- and SHH-activated medulloblastomas sensitive to cisplatin. Neuro-Oncol. 2023;25:899–912. 10.1093/neuonc/noac243 PMC10158119 36273330

[31] Dasari S, Tchounwou P. Cisplatin in cancer therapy: molecular mechanisms of action. Eur J Pharmacol. 2014;740:364–78. 10.1016/j.ejphar.2014.07.025 PMC4146684 25058905

[32] Zhao W, Steinfeld J, Liang F, Chen X, Maranon D, Jian Ma C, et al. BRCA1-BARD1 promotes RAD51-mediated homologous DNA pairing. Nature. 2017;550:360–5. 10.1038/nature24060 PMC5800781 28976962

[33] Saintigny Y, Delacôte F, Boucher D, Averbeck D, Lopez B. XRCC4 in G1 suppresses homologous recombination in S/G2, in G1 checkpoint-defective cells. Oncogene. 2007;26:2769–80. 10.1038/sj.onc.1210075 17057732

[34] Callen E, Zong D, Wu W, Wong N, Stanlie A, Ishikawa M, et al. 53BP1 enforces distinct pre- and post-resection blocks on homologous recombination. Mol cell. 2020;77:26–38.e7. 10.1016/j.molcel.2019.09.024 PMC6993210 31653568

[35] Chen C, Feng W, Lim P, Kass E, Jasin M. Homology-directed repair and the role of BRCA1, BRCA2, and related proteins in genome integrity and cancer. Annu Rev Cancer Biol. 2018;2:313–36. 10.1146/annurev-cancerbio-030617-050502 PMC6193498 30345412

[36] Ivanov D, Coyle B, Walker D, Grabowska A. In vitro models of medulloblastoma: choosing the right tool for the job. J Biotechnol. 2016;236:10–25. 10.1016/j.jbiotec.2016.07.028 27498314

