# Supplementary material for: KIF14 silencing suppresses cell proliferation and enhances chemosensitivity by modulating 53BP1 nucleocytoplasmic shuttling in non-WNT/non-SHH medulloblastoma
Source: Cell Death Discov. 2026 Jul 14;12:407. doi: 10.1038/s41420-026-03252-2 (PMC13645811; doi:10.1038/s41420-026-03252-2)
Supplement: Supplementary file 2 [file 41420_2026_3252_MOESM2_ESM.docx]

**Supplementary Table 1. The shRNA and siRNA sequences**

| **Gene** | **Primer** | **Sequence (5’-3’)** |
| --- | --- | --- |
| si*NC* | sense | TTCTCCGAACGTCACGT |
|  | antisense | ACGTGACGTTCGGAGAA |
| si*KIF14-1* | sense | GCCCGUUUAAUAGUCAACA |
|  | antisense | UGUUGACUAUUAAACGGGC |
| si*KIF14-2* | sense | CAGCGGUGAUAUUCUUGAUTT |
|  | antisense | AUCAAGAAUAUCACCGCUGTT |
| sh*KIF14* |  | GCCCGUUUAAUAGUCAACA |

**Supplementary Table 2. Primers for RT-qPCR**

| **Gene** | **Primer** | **Sequence (5′-3′)** |
| --- | --- | --- |
| *KIF14* | Forward | CCGACATTACAGATGCACCA |
|  | Reverse | CTTCATTCCTAAGCCTACACC |
| *β-Actin* | Forward | CATGTACGTTGCTATCCAGGC |
|  | Reverse | CTCCTTAATGTCACGCACGAT |

**Fig.S1 Kaplan–Meier survival curves comparing low and high KIF14 expression groups across all subtypes (A), WNT (B), and SHH (C) subgroups of medulloblastoma.**

**Fig.S2 Validation of KIF14 knockdown efficiency.** KIF14 expression levels were analyzed by RT-qPCR (A) and Western blot (B) in D283 and D341 cells transfected with siRNAs targeting KIF14.

**Fig.S3 Cleaved-caspase 3 immunochemistry of mice brain showed the tumor and proliferating cells in each treatment group.**
