# Supplementary material for: KIF14 silencing suppresses cell proliferation and enhances chemosensitivity by modulating 53BP1 nucleocytoplasmic shuttling in non-WNT/non-SHH medulloblastoma
Source: Cell Death Discov. 2026 Jul 14;12:407. doi: 10.1038/s41420-026-03252-2 (PMC13645811; doi:10.1038/s41420-026-03252-2)
Supplement: Supplementary file 3 [file 41420_2026_3252_MOESM1_ESM.docx]

**Figure 3A**

**Figure 4G**

**Figure 5L**

**Figure S2B**

β-actin
